# Risk factors for acute postoperative hypertension in non-cardiac major surgery: a case control study

**DOI:** 10.1186/s12871-023-02121-0

**Published:** 2023-05-16

**Authors:** Yaqing Zhou, Dongxue Luo, Luyi Shao, Zichuan Yue, Min Shi, Jie Zhang, Kangli Hui, Jingwei Xiong, Manlin Duan

**Affiliations:** 1 Department of Anesthesiology, Nanjing BenQ Medical Center, The Affiliated BenQ Hospital of Nanjing Medical University, Nanjing, Jiangsu 210019 China; 2 College of Anesthesiology, Xuzhou Medical University, Xuzhou, Jiangsu 221004 China; 3 Department of Anesthesiology, Affiliated Jinling Hospital, Medical School of Nanjing University, Nanjing, Jiangsu 210002 China

**Keywords:** Acute postoperative hypertension, Anesthesia recovery period, Post-anesthesia care unit, Non-cardiac major surgery, General anesthesia, Risk factors, Perioperative management

## Abstract

**Purpose:**

Acute postoperative hypertension (APH) is a common complication during the anesthesia recovery period that can lead to adverse outcomes, including cardiovascular and cerebrovascular accidents. Identification of risk factors for APH will allow for preoperative optimization and appropriate perioperative management. This study aimed to identify risk factors for APH.

**Patients and methods:**

In this retrospective single-center study, 1,178 cases were included. Data was entered by two investigators, and consistency analysis was performed by another. Patients were divided into APH and non-APH groups. A predictive model was built by multivariate stepwise logistic regression. The predictive ability of the logistic regression model was tested by drawing the receiver operating characteristic (ROC) curve and calculating the area under the curve (AUC). Hosmer and Lemeshow goodness-of-fit (GOF) test was performed to reflect the goodness of fit of the model. Calibration curve was created to represent the relationship between predicted risk and observed frequency. Sensitivity analysis was performed to evaluate the robustness of the results.

**Results:**

Multivariate logistic regression analysis showed that age over 65 years (OR = 3.07, 95% CI: 2.14 ~ 4.42, *P* < 0.001), female patients (OR = 1.37, 95% CI: 1.02 ~ 1.84, *P* = 0.034), presence of intraoperative hypertension (OR = 2.15, 95% CI: 1.57 ~ 2.95, *P* < 0.001), and use of propofol in PACU (OR = 2.14, 95% CI: 1.49 ~ 3.06, *P* < 0.001) were risk factors for APH. Intraoperative use of dexmedetomidine (OR = 0.66, 95% CI: 0.49 ~ 0.89, *P* = 0.007) was a protective factor. Higher baseline SBP (OR = 0.90, 95% CI: 0.89 ~ 0.92, *P* < 0.001) also showed some correlation with APH.

**Conclusions:**

The risk of acute postoperative hypertension increased with age over 65 years, female patients, intraoperative hypertension and restlessness during anesthesia recovery. Intraoperative use of dexmedetomidine was a protective factor for APH.

**Supplementary Information:**

The online version contains supplementary material available at 10.1186/s12871-023-02121-0.

## Introduction

The recovery period of anesthesia is an important link of the recovery from the anesthesia state to the normal physiological state, including the depth of anesthesia, gradual recovery of sensory and motor function, spontaneous breathing and gradual maintenance of normal breathing, respiratory reflex recovery and waking. The awakening period of anesthesia is a more dangerous perioperative period, during which patients are at risk of multiple complications. Acute postoperative hypertension (acute postoperative hypertension, APH) is a common complication in patients during the awakening period of anesthesia. Patient developing APH during the recovery period of general anesthesia may lead to serious postoperative adverse events, increasing the perioperative risk. The main hazards include: (1) increasing myocardial oxygen consumption, resulting in myocardial ischemia, arrhythmia, and even myocardial infarction [1]; (2) leading to surgical site bleeding, anastomotic cracking, local hematoma [2]; (3) induced cerebrovascular spasm, thrombosis or cerebrovascular rupture, hemorrhage, stroke [3], etc.

However, it became clear that it is very important to accurately determine the epidemiology of APH in a population of patients undergoing major general anesthesia non-cardiac surgery and to identify all risk factors for APH. The purpose of this study was to explore risk factors for APH in patients recovering from general anesthesia. Anesthesiologists can develop a targeted anesthesia management plan according to common factors influencing elevated blood pressure in patients recovering from anesthesia, thereby improving perioperative anesthesia management and reducing complications.

## Methods and materials

### Study design and participants

**Fig. 1:**
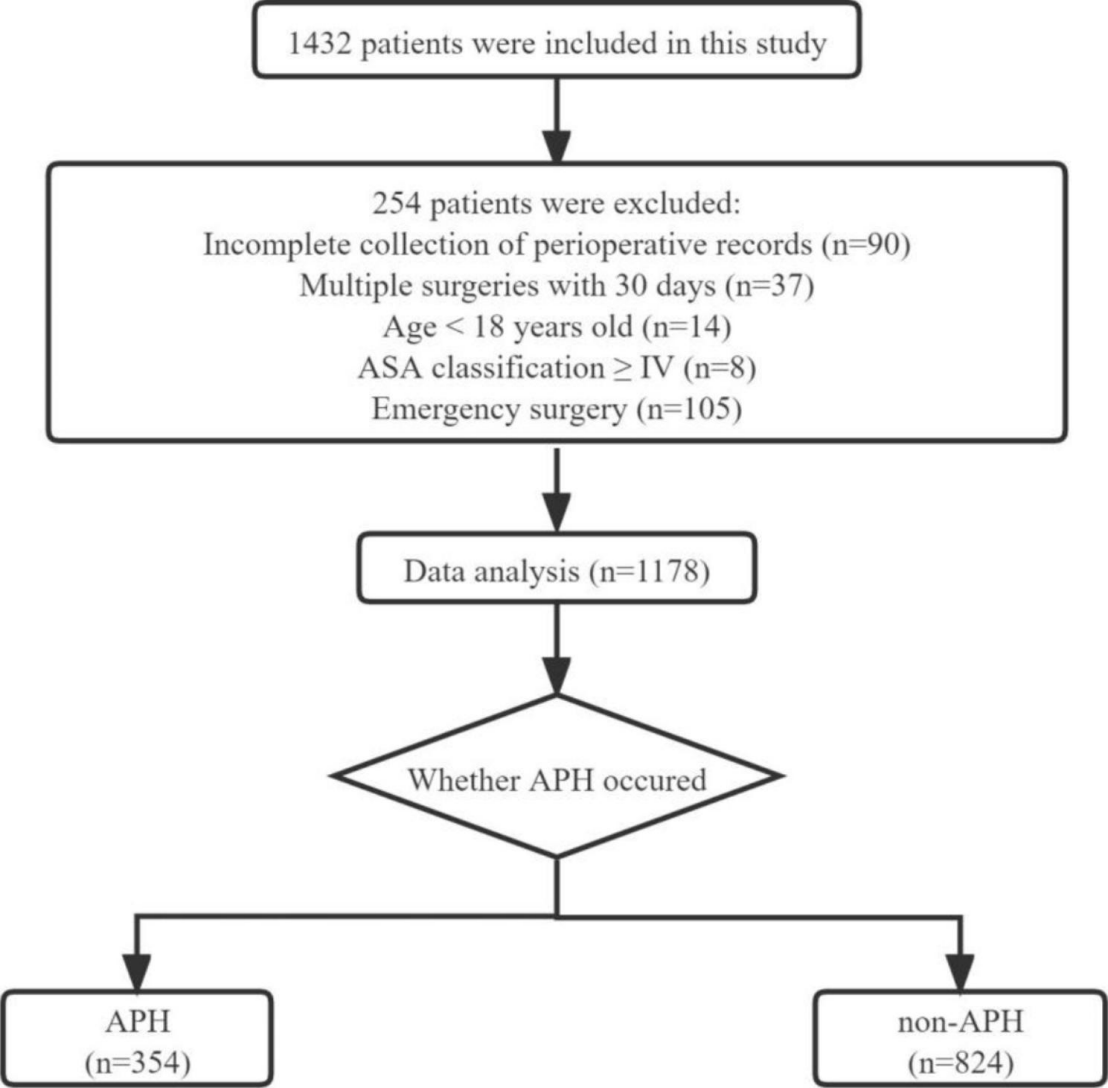
Flowchart

This was a single-center, retrospective, case control study conducted at Jinling Hospital, Medical School of Nanjing University and approved by the Ethics Committee, including a waiver of informed consent (approval number: 2022NZKY-010-01). Patients who underwent major non-cardiac surgery under general anesthesia between September 2020 and October 2020 and entered the post-anesthesia care unit (PACU) to recover from anesthesia were selected. Patients were divided into APH and non-APH groups according to the occurrence of APH events during the anesthesia recovery period (Fig. 1).

Inclusion criteria are as follows. (1) patients undergoing major non-cardiac surgery under general anesthesia, and (2) patients admitted to the PACU for recovery from anesthesia after surgery.

Exclusion criteria were as follows. (1) Incomplete perioperative hemodynamic record collection, (2) New York Heart Association (NYHA) grade III or higher, (3) other surgery within 30 days prior to surgery, (4) age < 18 years, and (5) American Society of Anesthesiologists (ASA) classification IV or higher, (6) Patients undergoing emergency surgery.

### Sample size determination

We pre-collected analyzed information on approximately 400 patients and found that 14 factors were associated with APH. The incidence of APH at the time of anesthesia recovery was approximately 20%. Dividing the number of risk factors included in the multivariate logistic regression by the incidence of APH and multiplying by 10 yielded a required sample size of approximately 700. In order to analyze as many patients with APH during the anesthesia recovery period as possible, we ultimately included 1178 patients.

### Definition of APH

APH was defined as an increase in systolic blood pressure greater than 20% of baseline blood pressure or diastolic blood pressure greater than 110 mmHg during anesthesia recovery [4]. Blood pressure measured with an oscillometric electronic blood pressure monitor at rest on admission was used as the baseline blood pressure.

### Anesthesia management

Anesthesia induction drugs were propofol, sufentanil, midazolam, rocuronium or cisatracurium, and anesthesia maintenance drugs were propofol, sevoflurane, remifentanil, and cisatracurium. Intraoperative arterial blood pressure monitoring was performed in all patients, and volume management was performed according to intraoperative hemodynamic indicators. Patients were transported directly to the PACU or intensive care unit (ICU) by transport stretcher almost immediately after surgery. Management was provided by the same team of anesthesiologists during the recovery period. When necessary, analgesic, propofol, flumazenil and neostigmine were used to ensure smooth recovery during the recovery period. After extubation, all patients received oxygen with nasal cannula. During the anesthesia recovery period, blood pressure was monitored with a cuff every 5 min after admission to the PACU and before leaving the PACU.

### Data collection

Patients’ personal information, medical history, and postoperative related information were collected in an electronic medical record system. Relevant information during the intraoperative and recovery periods was collected by the Anesthesia Clinical Information System, and the last examination index before surgery was collected by the Examination System.

Data was entered by two investigators. Each constructed an EpiData database (V3.1, EpiData Association, Odense, Denmark). Discrepancies were verified and corrected by third-party participants. Finally, the database is exported in the form of Excel tables.

### Statistical analysis

The normality of the distributions was confirmed by graphs and the Shapiro-Wilk test. Quantitative variables were denoted by mean ± standard deviation or median (interquartile range), depending on the distribution. Qualitative variables were expressed as numbers (%).

All observed indicators were subjected to univariate logistic regression analysis, factors with *P*-values < 0.1 in univariate logistic regression were entered into a multivariate logistic regression, and independent risk factors for APH were determined by stepwise regression methods. Finally, for all variables entering the model, a diagnosis of multicollinearity was made using the Variance Inflation Factor (VIF). Finally, variables with *P*-values < 0.1 were entered in a multivariate binary logistic regression model. ORs and their 95% CIs were calculated for the strength of the association between each observed indicator and APH. The predictive ability of the logistic regression model was tested by drawing the receiver operating characteristic (ROC) curve and calculating the area under the curve (AUC). Hosmer and Lemeshow goodness-of-fit (GOF) test was performed to reflect the goodness of fit of the model. Calibration curve was created to better represent the relationship between predicted risk and observed frequency.

### Sensitivity analysis

To assess the robustness of our model and conclusions, we performed a sensitivity analysis. Excluding the baseline blood pressure, the parameters with *P*-values < 0.1 in the univariate logistic regression analysis were reintroduced into the multivariate logistic regression model.

All statistical tests were two-tailed, with *P*-values < 0.05 considered statistically significant. Analyses were performed using R (version 4.1.2, the R Foundation for Statistical Computing, http://www.r-project.org.)

## Results

Tables 1 and 2 show the basic characteristics and perioperative data for all patients, respectively. All risk factors for APH were imputed by univariate logistic regression. As a result, we found 10 variables with *P* < 0.1 among all risk factors (Table 3).

**Table 1:** Basic characteristics of all patients

Variables	Overall (n = 1178)	non-APH (824)	APH (n = 354)
Age ≥ 65, n(%)	258 (21.9%)	161 (19.5%)	97 (27.4%)
Male / Female	602 / 576	443 / 381	159 / 195
Obesity^a^, n(%)	115 (9.8%)	84 (10.2%)	31 (8.8%)
Baseline SBP, mmHg	126.30 ± 26.58	130.46 ± 29.97	116.63 ± 11.27
Baseline DBP, mmHg	76.46 ± 9.93	78.18 ± 10.00	72.45 ± 8.52
Smoking, n(%)	177 (15.0%)	128 (15.5%)	49 (13.8%)
Drinking, n(%)	134 (11.4%)	99 (12.0%)	35 (9.9%)
Stroke, n(%)	81 (6.9%)	54 (6.6%)	27 (7.6%)
CHD, n(%)	35 (3.0%)	27 (3.3%)	8 (2.3%)
Diabetes, n(%)	136 (11.5%)	92 (11.2%)	44 (12.4%)
Hypertension, n(%)	323 (27.4%)	234 (28.4%)	89 (25.1%)
Other diseases^b^, n(%)	66 (5.6%)	49 (5.9%)	17 (4.8%)
ASA classification, n(%)			
I	100 (8.5%)	73 (8.9%)	27 (7.6%)
II	1019 (86.5%)	715 (86.8%)	304 (85.9%)
III	59 (5.0%)	36 (4.4%)	23 (6.5%)
Laboratory examination			
Glu, mmol/L	5.74 ± 1.92	5.73 ± 1.92	5.77 ± 1.93
Hb, g/L	129.20 ± 19.50	130.15 ± 19.94	127.00 ± 18.28
Medication history, n(%)			
GC	18 (1.5%)	14 (1.7%)	4 (1.1%)
NSAIDs	19 (1.6%)	16 (1.9%)	3 (0.8%)
Antihypertensives	336 (28.5%)	238 (28.9%)	98 (27.7%)

Abbreviations: SBP, systolic blood pressure; DBP, diastolic blood pressure; Glu, blood glucose; UA, blood uric acid; CHD, coronary artery disease; GC, glucocorticoids; NSAIDs, nonsteroidal anti-inflammatory drugs

^a^Obesity: BMI ≥ 28 kg/m^2^, ^b^Other diseases: chronic glomerulonephritis, vasculopathy, pheochromocytoma, Cushing’s syndrome, polycystic ovary syndrome, hyperthyroidism, hypothyroidism, primary aldosteronism, pregnancy-induced hypertension, purpura, meningioma, anxiety, depression

**Table 2:** Perioperative data of all patients

Variables	Overall (n = 1178)	non-APH (824)	APH (n = 354)
Surgery, n(%)			
Brain	130 (11.0%)	96 (11.7%)	34 (9.6%)
Head and neck	134 (11.4%)	96 (11.7%)	38 (10.7%)
Chest	31 (2.6%)	21 (2.5%)	10 (2.8%)
Abdomen	626 (53.1%)	424 (51.5%)	202 (57.1%)
Limbs	139 (11.8%)	99 (12.0%)	40 (11.3%)
Spine	118 (10.0%)	88 (10.7%)	30 (8.5%)
Airway, n(%)			
Endotracheal tube	926 (78.6%)	641 (77.8%)	285 (80.5%)
Laryngeal mask airway	252 (21.4%)	183 (22.2%)	69 (19.5%)
Intraoperative hypertension^a^, n(%)	347 (29.5%)	213 (25.8%)	134 (37.9%)
Perioperative drugs			
Propofol, mg	1100.00 [700.00, 1300.00]	1095.00 [700.00, 1300.00]	1100.00 [700.00, 1300.00]
Sevoflurane, mL	0.00 [0.00, 20.00]	0.00 [0.00, 20.00]	0.00 [0.00, 20.00]
Sufentanil, ug	35.00 [35.00, 40.00]	35.00 [35.00, 40.00]	35.00 [35.00, 40.00]
Remifentanil, mg	1.01 ± 0.42	1.01 ± 0.41	1.03 ± 0.45
Midazolam, mg	2.00 [2.00, 3.00]	2.00 [2.00, 3.00]	2.00 [2.00, 3.00]
Cisatracurium, mg	20.00 [20.00, 35.00]	20.00 [20.00, 35.00]	25.00 [20.00, 35.00]
Etomidate, mg	0.00 [0.00, 10.00]	0.00 [0.00, 10.00]	0.00 [0.00, 10.00]
Dexmedetomidine, n(%)	483 (41.0%)	356 (43.2%)	127 (35.9%)
Rocuronium, n(%)	693 (58.8%)	482 (58.5%)	211 (59.6%)
Flurbiprofen axetil, n(%)	872 (74.0%)	611 (74.2%)	261 (73.7%)
Parecoxib, n(%)	136 (11.5%)	92 (11.2%)	44 (12.4%)
Intraoperative vasoactive agent, n(%)			
Hypertensive agent	10 (0.8%)	9 (1.1%)	1 (0.3%)
Antihypertensives	829 (70.4%)	577 (70.0%)	252 (71.2%)
None	339 (28.8%)	238 (28.9%)	101 (28.5%)
Intraoperative net intake^b^, mL	839.23 ± 395.31	833.12 ± 381.44	853.43 ± 426.07
Transfusion, n(%)	77 (6.5%)	48 (5.8%)	29 (8.2%)
Surgery time, min	144.39 ± 85.84	144.27 ± 85.05	144.69 ± 87.78
Urethral catheter, n(%)	981 (83.3%)	671 (81.4%)	310 (87.6%)
PACU drugs, n(%)			
Analgesic	29 (2.5%)	15 (1.8%)	14 (4.0%)
Propofol	226 (19.2%)	130 (15.8%)	96 (27.1%)
Neostigmine	114 (9.7%)	77 (9.3%)	37 (10.5%)
Flumazenil	139 (11.8%)	95 (11.5%)	44 (12.4%)
Duration of PACU, min	98.21 (31.91)	96.02 (31.43)	103.31 (32.46)

^a^ Intraoperative hypertension was defined as systolic blood pressure greater than 140 mmHg or diastolic blood pressure greater than 90 mmHg during surgery. ^b^Intraoperative net intake was defined as the value of the infusion minus the loss during the surgery

**Table 3:** Univariate and multivariate analysis of risk factors associated with APH

Variables	Univariate analysis		Multivariate analysis	
	OR (95%CI)	*P*	OR (95%CI)	*P*
Age ≥ 65	1.55(1.16,2.07)	0.003	3.07(2.14,4.42)	< 0.001
Sex-Female	1.43(1.11,1.83)	0.005	1.37(1.02,1.84)	0.034
Baseline SBP	0.92(0.91,0.93)	< 0.001	0.90(0.89,0.92)	< 0.001
Baseline DBP	0.93(0.92,0.95)	< 0.001	-	-
Hb	0.99(0.99,1.00)	0.011	-	-
Intraoperative hypertension^a^	1.75(1.34,2.28)	< 0.001	2.15(1.57,2.95)	< 0.001
Dexmedetomidine	0.74(0.57,0.95)	0.019	0.66(0.49,0.89)	0.007
Urethral catheter	1.61(1.13,2.33)	0.010	-	-
Analgesic in PACU	2.22(1.05,4.67)	0.034	-	-
Propofol in PACU	1.99(1.47,2.68)	< 0.001	2.14(1.49,3.06)	< 0.001

Variables were considered significant if *P* < 0.1 on univariate analysis. 10 variables were statistically significant. ^a^ Intraoperative hypertension was defined as systolic blood pressure greater than 140 mmHg or diastolic blood pressure greater than 90 mmHg during surgery

Abbreviations: SBP, systolic blood pressure; DBP, diastolic blood pressure

As a rule, variables with *P* < 0.1 were included in the multivariate stepwise logistic regression. As shown in the Table 3, relatively high baseline systolic blood pressure (OR = 0.90, 95% CI: 0.89 ~ 0.92, *P* < 0.001) and intraoperative use of dexmedetomidine (OR = 0.66, 95% CI: 0.49 ~ 0.89, *P* = 0.007) may reduce the occurrence of acute postoperative hypertension, while age over 65 years (OR = 3.07, 95% CI: 2.14 ~ 4.42, *P* < 0.001), female patients (OR = 1.37, 95% CI: 1.02 ~ 1.84, *P* = 0.034), occurrence of intraoperative hypertension (OR = 2.15, 95% CI: 1.57 ~ 2.95, *P* < 0.001), and propofol use in the PACU (OR = 2.14, 95% CI: 1.49 ~ 3.06, *P* < 0.001) appear to be positively associated with APH.

**Fig. 2:**
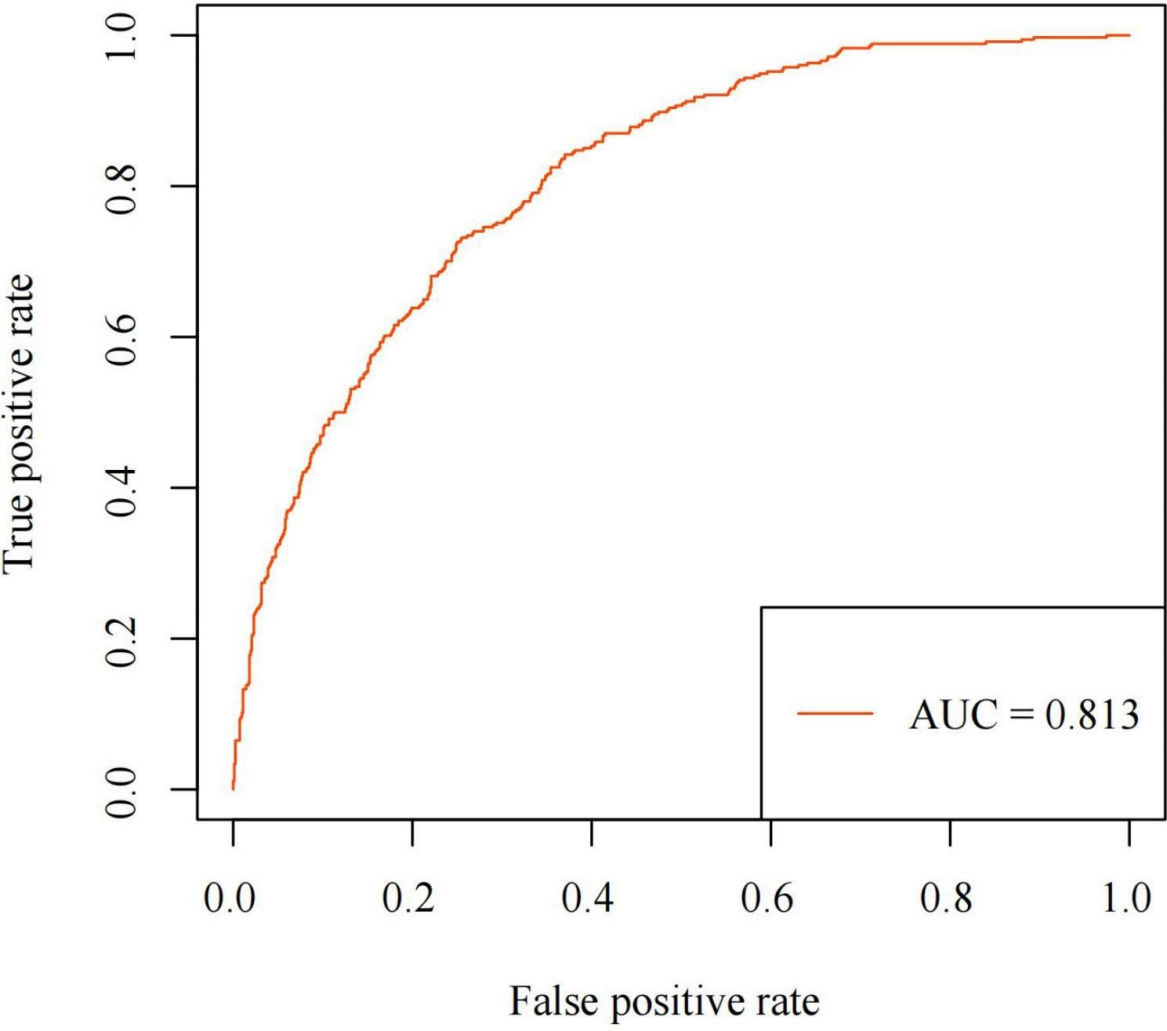
Receiver operating characteristic curve (ROC curve)

ROC curve was used to validate the discriminability of the model. The ROC curve represents the ability of selected risk factors to discriminate between non-APH and APH. The AUC obtained was 0.813 (Fig. 2), indicating that the model has good discriminability.

**Fig. 3:**
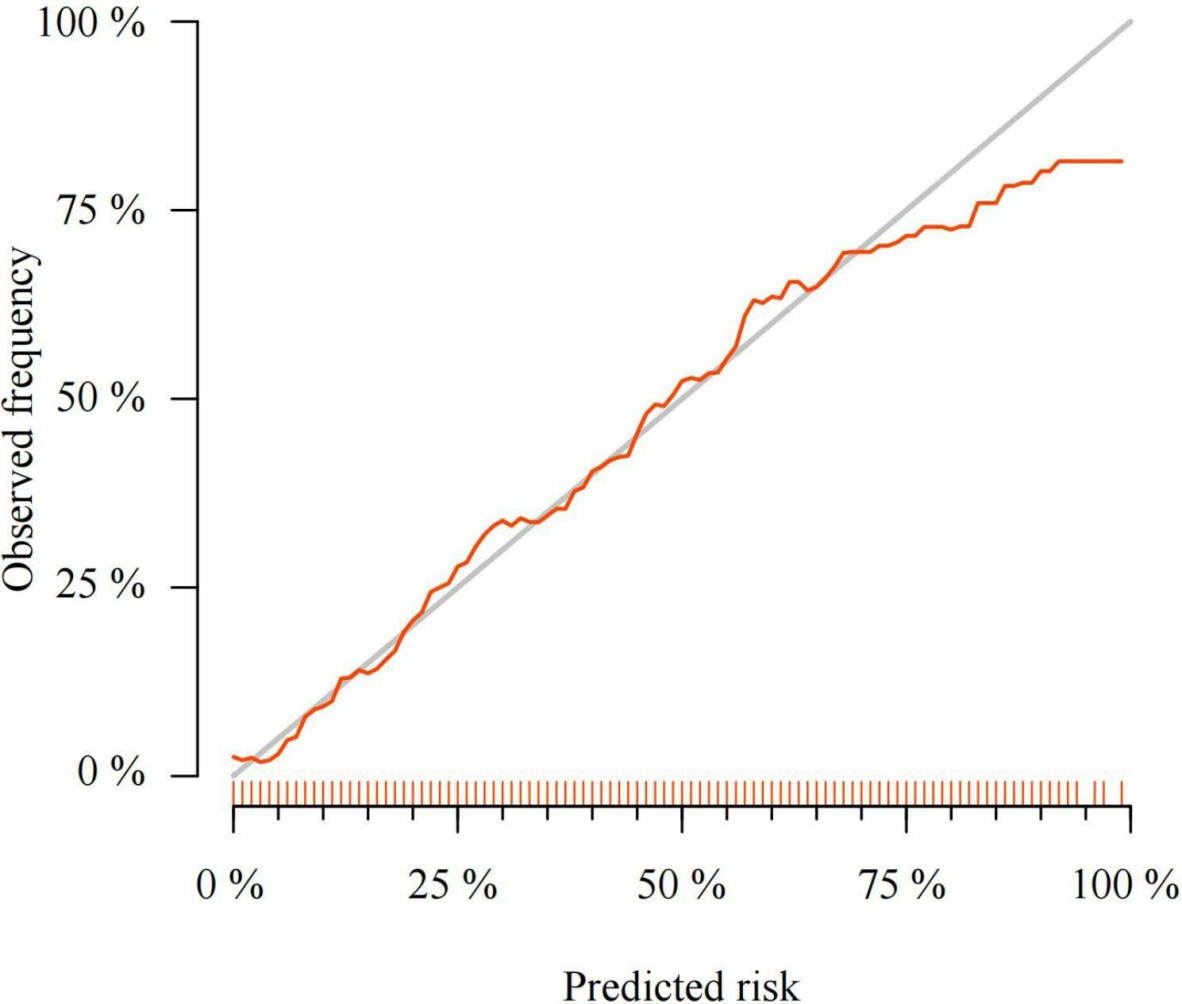
Calibration curve

The results of the Hosmer and Lemeshow GOF test was performed, with *P*-values = 0.387. The difference between the predicted model and the observed values is not statistically significant, indicating a good fit. The calibration curve corresponds to the diagonal line (Fig. 3). This indicates a high degree of goodness of fit.

## Discussion

To date, there is no accepted definition of APH. Therefore, the reported incidence of APH depends on the criteria used and the type of surgery. In the present study, we found that the incidence of APH was 30.1%.

There are many factors that cause APH in patients, but no consensus has emerged. In the present study, analysis confirmed that age is one of the most important factors in the occurrence of APH in patients. One study showed that the prevalence of hypertension increases from 8% in those aged 18 to 39 years to 65% in those aged 60 years and older [5]. In this study, there were 258 patients (21.9%) over the age of 65 years, of whom 97 patients had APH, and the incidence of APH in the elderly was as high as 37.6%. On the one hand, aging is seen as a major factor, since cardiovascular function in the elderly gradually declines with increasing age and disease progression. On the other hand, 137 (53.1%) of the elderly in this study had a history of hypertension. On May 6, 2020, the International Society of Hypertension (ISH) published its own global hypertension practice guidelines, dividing hypertensive patients into three categories [6]. Among them, high-risk patients have a very low tolerance for stress responses to various adverse stimuli. In elderly patients with primary hypertension, arterial pressure acts extraordinarily long in response to stimuli that cause vasoconstriction, such as pain and hypoxemia. Then, a sudden and severe rise in blood pressure may occur during recovery from anesthesia [7, 8], which is in good agreement with the present results.

The results of this study showed that the use of propofol in PACU was significantly associated with the occurrence of APH. In 2017, an online survey initiated by The German Society of Anesthesiologists showed that experienced anesthesiologists tend to use propofol to treat restlessness during the recovery period [9]. Kim et al. suggested that propofol can prevent or alleviate restlessness [10]. In fact, in our study center, anesthesiologists tend first to use propofol to control patients’ restlessness symptoms quickly, avoid adverse effects caused by restlessness, and then comprehensively analyze the patient’s condition and provide other corresponding and appropriate treatments. Therefore, in this study, we believe that adding propofol during the recovery period implies the development of restlessness. Since restlessness can be a stimulus for hypertension [11], patients who received additional propofol were more likely to develop APH. We suggest that restlessness be addressed promptly to reduce the incidence of APH.

It can be seen that patients with higher baseline blood pressure were less likely to develop APH, but this result may be related to the definition of APH in this study. The study noted that patients with intraoperative hypertension were more likely to develop APH than patients without intraoperative hypertension, which seems reasonable. Intraoperative hypertension often indicates that the patient was in a state of sympathetic agitation, elevated catecholamine levels, or volume overload during surgery. If these conditions are not addressed and corrected in a timely manner, patients are often more likely to develop hypertension postoperatively.

This study found that compared with male patients, APH was more likely to occur in female patients. With increasing age, the degree of increase in sympathetic nerve activity that causes vasoconstriction is higher in women than in men [12]. This sex difference may be related to estrogen activity levels. Among the 576 female patients included in this study, about 337(59%) patients were older than 50 years old, and the proportion of women with menopause was larger. Postmenopausal women experience changes in estrogen levels and are prone to emotional agitation, resulting in increased excitability of sympathetic nervous system and changes in endocrine function, thus easily leading to abnormal increase in blood pressure [13].

In this study, the role of dexmedetomidine on APH was examined and found to have a certain protective effect. A subgroup analysis of the dexmedetomidine-treated (Dex group) and non-treated (non-Dex group) groups was performed.The results showed that the incidence of APH was statistically lower in the Dex group (Additional file 1). Studies have demonstrated that perioperative infusion of dexmedetomidine results in lower blood pressure during anesthesia recovery [14, 15]. It has been reported in the literature that intraoperative ues of dexmedetomidine reduces the stress response of patients during tracheal intubation and extubation [16], and Turan et al. [17] found that the use of dexmedetomidine in neurosurgical procedures improved the quality of extubation, stabilized patient hemodynamics during extubation. They were consistent with the results obtained in the present study.

In the sensitivity analysis, a stepwise logistic regression was performed using the model excluding the baseline blood pressure. The results were compared with the primary analysis. The results did not change significantly, suggesting that our results are fairly robust (Additional file 2).

This study is a retrospective study and has unavoidable limitations. This study has some flaws in the process of extracting clinical information for patients in recovery. Data collection is every 5 min, which may result in missing important data, and the inclusion of relevant risk factors in the PACU is not sufficient. For patients whose blood pressure was controlled with oral antihypertensive drugs over a long period of time, it was not possible to collect relevant information because the type and method of medication were not recorded in detail in the medical record system. Therefore, it remains necessary to conduct a prospective study in the later stages of the disease to record detailed clinical information on patients’ baseline data and recovery periods in order to improve the predictive model.

In conclusion, the risk of acute postoperative hypertension was found to be increased with age ≥ 65 years, female patients, restlessness during recovery and intraoperative hypertension. Intraoperative use of dexmedetomidine was protective factors for APH. These findings may have implications for postoperative blood pressure management in patients admitted to the PACU.

## Electronic supplementary material

Below is the link to the electronic supplementary material.

Supplementary Material 1

Supplementary Material 2

## Data Availability

All data generated or analysed during this study are included in this published article and its supplementary information files.

## Abbreviations

APH: Acute postoperative hypertension
BMI: Body mass index
ASA: American Society of Anesthesiologists
PACU: Post-anesthesia care unit
NSAIDs: Nonsteroidal anti-inflammatory drugs
NYHA: New York Heart Association
ICU: Intensive care unit
OR: Odds ratio
CI: Confidence interval
ROC: Receiver operating characteristic
AUC: Area under the curve
GOF: Goodness-of-fit
S.E: Standard error

## References

[1] Fayad AA, Yang HY, Ruddy TD (2011). Perioperative myocardial ischemia and isolated systolic hypertension in non-cardiac surgery. Can J Anaesth.

[2] Guay J (2006). Postoperative pain significantly influences postoperative blood loss in patients undergoing total knee replacement. Pain Med.

[3] Mashour GA, Shanks AM, Kheterpal S (2011). Perioperative stroke and associated mortality after noncardiac, nonneurologic surgery. Anesthesiology.

[4] Marik PE, Varon J (2009). Perioperative hypertension: a review of current and emerging therapeutic agents. J Clin Anesth.

[5] Wang Y, Wang QJ (2004). The prevalence of prehypertension and hypertension among US adults according to the new joint national committee guidelines: new challenges of the old problem. Arch Intern Med.

[6] Unger T, Borghi C, Charchar F, Khan NA, Poulter NR, Prabhakaran D (2020). 2020 International Society of Hypertension Global Hypertension Practice Guidelines. Hypertension.

[7] Yu J, Hu J, Huang C, Ying M, Peng X, Wei H (2013). The impact of age and comorbidity on postoperative complications in patients with advanced gastric cancer after laparoscopic D2 gastrectomy: results from the chinese laparoscropic gastrointestinal surgery study (CLASS) group. Eur J Surg Oncol.

[8] Wen W, Luo R, Tang X, Tang L, Huang HX, Wen X (2015). Age-related progression of arterial stiffness and its elevated positive association with blood pressure in healthy people. Atherosclerosis.

[9] Huett C, Baehner T, Erdfelder F, Hoehne C, Bode C, Hoeft A (2017). Prevention and Therapy of Pediatric Emergence Delirium: A National Survey. Paediatr Drugs.

[10] Kim MS, Moon BE, Kim H, Lee JR (2013). Comparison of propofol and fentanyl administered at the end of anaesthesia for prevention of emergence agitation after sevoflurane anaesthesia in children. Br J Anaesth.

[11] Cole JW, Murray DJ, McAllister JD, Hirshberg GE (2002). Emergence behaviour in children: defining the incidence of excitement and agitation following anaesthesia. Paediatr Anaesth.

[12] Best SA, Okada Y, Galbreath MM, Jarvis SS, Bivens TB, Adams-Huet B (2014). Age and sex differences in muscle sympathetic nerve activity in relation to haemodynamics, blood volume and left ventricular size. Exp Physiol.

[13] Wyatt SB, Akylbekova EL, Wofford MR, Coady SA, Walker ER, Andrew ME (2008). Prevalence, awareness, treatment, and control of hypertension in the Jackson Heart Study. Hypertension.

[14] Rekatsina M, Theodosopoulou P, Staikou C (2021). Effects of Intravenous Dexmedetomidine Versus Lidocaine on Postoperative Pain, analgesic consumption and functional recovery after abdominal gynecological surgery: a randomized placebo-controlled double blind study. Pain Physician.

[15] Choi JW, Joo JD, Kim DW, In JH, Kwon SY, Seo K (2016). Comparison of an intraoperative infusion of Dexmedetomidine, Fentanyl, and Remifentanil on Perioperative Hemodynamics, Sedation Quality, and Postoperative Pain Control. J Korean Med Sci.

[16] Mikawa K, Nishina K, Maekawa N, Obara H (1996). Comparison of nicardipine, diltiazem and verapamil for controlling the cardiovascular responses to tracheal intubation. Br J Anaesth.

[17] Turan G, Ozgultekin A, Turan C, Dincer E, Yuksel G (2008). Advantageous effects of dexmedetomidine on haemodynamic and recovery responses during extubation for intracranial surgery. Eur J Anaesthesiol.

