# Supplementary material for: Risk factors for acute postoperative hypertension in non-cardiac major surgery: a case control study
Source: BMC Anesthesiol. 2023 May 16;23:167. doi: 10.1186/s12871-023-02121-0 (PMC10186778; doi:10.1186/s12871-023-02121-0)
Supplement: Supplementary file 1 — Supplementary Material 1 [file 12871_2023_2121_MOESM1_ESM.docx]

**Additional file 1.** Subgroup analysis stratified by dexmedetomidine (n=1178)

| Variables | non-Dexmedetomidine  （n = 695） | Dexmedetomidine  (n =483) | *P* |
| --- | --- | --- | --- |
| Age ≥ 65, n(%) | 164 (23.6%) | 94 (19.5%) | 0.106 |
| Male / Female | 352/343 | 250/233 | 0.752 |
| Obesity^a^, n(%) | 61 (8.8%) | 54 (11.2%) | 0.205 |
| Baseline SBP, mmHg | 126.01 ± 15.76 | 126.72 ± 36.97 | 0.654 |
| Baseline DBP, mmHg | 76.43 ± 9.90 | 76.51 ± 9.98 | 0.893 |
| Smoking, n(%) | 110 (15.8%) | 67 (13.9%) | 0.400 |
| Drinking, n(%) | 78 (11.2%) | 56 (11.6%) | 0.917 |
| Stroke, n(%) | 46 (6.6%) | 35 (7.2%) | 0.763 |
| CHD, n(%) | 19 (2.7%) | 16 (3.3%) | 0.688 |
| Diabetes, n(%) | 78 (11.2%) | 58 (12.0%) | 0.747 |
| Hypertension, n(%) | 191 (27.5%) | 132 (27.3%) | 1.000 |
| Other diseases^b^, n(%) | 28 (4.0%) | 38 (7.9%) | 0.007 |
| ASA classification, n(%) |  |  | 0.001 |
| Ⅰ | 74 (10.6%) | 26 (5.4%) |  |
| Ⅱ | 580 (83.5%) | 439 (90.9%) |  |
| Ⅲ | 41 (5.9%) | 18 (3.7%) |  |
| Laboratory examination |  |  |  |
| Glu, mmol/L | 5.61 ± 1.83 | 5.93 ± 2.04 | 0.005 |
| Hb, g/L | 129.93 ± 18.71 | 128.17 ± 20.56 | 0.128 |
| Medication history, n(%) |  |  |  |
| GC | 12 (1.7%) | 6 (1.2%) | 0.671 |
| NSAIDs | 14 (2.0%) | 5 (1.0%) | 0.281 |
| Antihypertensives | 196 (28.2%) | 140 (29.0%) | 0.820 |
| APH, n (%) | 227 (32.7%) | 127 (26.3%) | 0.023 |

Categorical variables were evaluated with the chi-square test, and continuous variables were tested with Student's t-test or Mann-Whitney's U-test.

Abbreviations: SBP, systolic blood pressure; DBP, diastolic blood pressure; CHD，coronary heart disease; GC, glucocorticoid; NSAIDs, non-steroidal anti-inflammatory drugs; APH, acute postoperative hypertension.

^a^Obesity: BMI ≥ 28kg/m^2^, ^b^Other diseases: chronic glomerulonephritis, renovascular disease, pheochromocytoma, cushing syndrome, polycystic ovary syndrome, hyperthyroidism, hypothyroidism, primary aldosteronism, pregnancy-induced hypertension, purpura aura, meningioma, anxiety, depression.
