# Supplementary material for: Risk factors for acute postoperative hypertension in non-cardiac major surgery: a case control study
Source: BMC Anesthesiol. 2023 May 16;23:167. doi: 10.1186/s12871-023-02121-0 (PMC10186778; doi:10.1186/s12871-023-02121-0)
Supplement: Supplementary file 2 — Supplementary Material 2 [file 12871_2023_2121_MOESM2_ESM.docx]

**Additional file 2.** Results of sensitivity analysis

| Variable | β | S.E | OR（95%CI） | *P* |
| --- | --- | --- | --- | --- |
| Age ≥ 65 | 0.40 | 0.15 | 1.50(1.11,2.01) | 0.008 |
| Sex-Female | 0.37 | 0.13 | 1.45(1.12,1.88) | 0.005 |
| Intraoperative hypertension^a^ | 0.45 | 0.14 | 1.57(1.19,2.06) | 0.001 |
| Dexmedetomidine | -0.36 | 0.14 | 0.70(0.53,0.91) | 0.009 |
| Propofol in PACU | 0.65 | 0.16 | 1.91(1.40,2.60) | <0.001 |

Significant at *P* < 0.05. ^a^Intraoperative hypertension was defined as systolic blood pressure greater than 140 mmHg or diastolic blood pressure greater than 90 mmHg during surgery. Dexmedetomidine referred to intraoperative use of dexmedetomidine.

Abbreviations: S.E, standard error; OR, odds ratio; CI, confidence interval; OR, odds ratio; CI, confidence interva.
